# Parametric Effects of Fused Filament Fabrication Approach on Surface Roughness of Acrylonitrile Butadiene Styrene and Nylon-6 Polymer

**DOI:** 10.3390/ma15155206

**Published:** 2022-07-27

**Authors:** Ray Tahir Mushtaq, Asif Iqbal, Yanen Wang, Quentin Cheok, Saqlain Abbas

**Affiliations:** 1 Department of Industry Engineering, School of Mechanical Engineering, Northwestern Polytechnical University, Xi’an 710072, China; 2 Faculty of Integrated Technologies, Universiti Brunei Darussalam, Jalan Tungku Link, Gadong BE1410, Brunei; asif.asifiqbal@gmail.com (A.I.); quentin.cheok@ubd.edu.bn (Q.C.); 3 Department of Mechanical Engineering, University of Engineering and Technology, Lahore, Narowal Campus, Narowal 51600, Pakistan; saqlain.abbas@uet.edu.pk

**Keywords:** fused filament fabrication, additive manufacturing, Taguchi, ANOVA, surface roughness, polymer

## Abstract

This research objective is to optimize the surface roughness of Nylon-6 (PA-6) and Acrylonitrile Butadiene Styrene (ABS) by analyzing the parametric effects of the Fused Filament Fabrication (FFF) technique of Three-Dimensional Printing (3DP) parameters. This article discusses how to optimize the surface roughness using Taguchi analysis by the S/N ratio, ANOVA, and modeling methods. The effects of ABS parameters (initial line thickness, raster width, bed temperature, build pattern, extrusion temperature, print speed, and layer thickness) and PA-6 parameters (layer thickness, print speed, extrusion temperature, and build pattern) were investigated with the average surface roughness (Ra) and root-mean-square average surface roughness (Rq) as response parameters. Validation tests revealed that Ra and Rq decreased significantly. After the optimization, the Ra-ABS and Rq-PA-6 for the fabricated optimized values were 1.75 µm and 21.37 µm, respectively. Taguchi optimization of Ra-ABS, Rq-ABS, Ra-PA-6, and Rq-PA-6 was performed to make one step forward to use them in further research and prototypes.

## 1. Introduction

**Figure 1:**
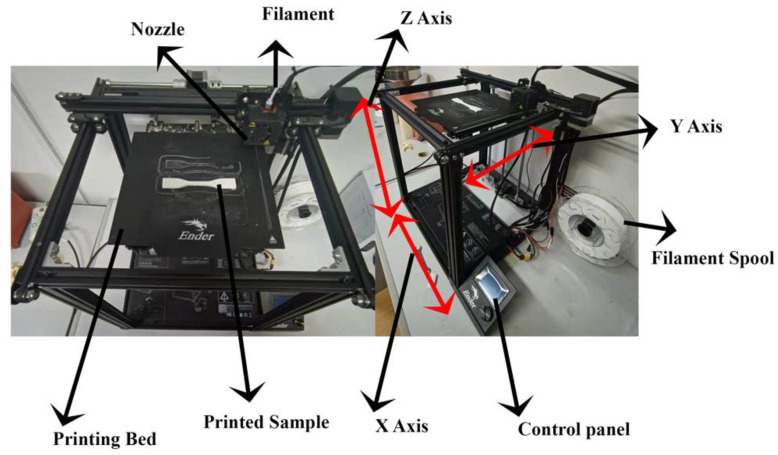
Working of the FFF-based 3D printer.

The term “Additive Manufacturing” (AM) states the process of joining material successively layer-by-layer to fabricate the object [1]. The material component is constructed layer after layer [2]. The most common AM techniques are sheet lamination, directed energy deposition, vat polymerization, binder jetting, and selective laser machining [3]. Crump [4] published a patent on the Fused Filament Fabrication (FFF) technique of Three-Dimensional Printing (3DP) around 1988. Figure 1 depicts the scheme of the FFF printer’s operation.

Various FFF implementations exist in several industries, including the emergency reaction to Coronavirus Disease 2019 (COVID-19), during which 3DP acted as mobile factories and assisted in quick fabrication [5]. Numerous engineering and industry disciplines, including medical implants, dentistry, aviation, refrigeration systems, and automotive goods, benefit from technology through various applications [6,7]. Dental models made using additive manufacturing technologies such as FFF and PolyJet were proven accurate and exact [8,9]. The authors [10] studied personalized prosthetic devices, which resulted in a rise in the efficiency of the current production process. According to the substantial literature, FFF 3DP is used to build fiber composite structures for automobile and aerospace, conductive structures, prosthetics [11], biological and construction applications, biochemical products, medical products [12,13], jewelry industries, soot particles filters, lightweight heating elements, and patterns for investment artifacts [14]. FFF 3DP is widely used to optimize the qualities of many materials, particularly the tensile strength of the material [15], auto parts [16], prototypes fabrication for research [17,18,19], micro-structural investigation [20,21], and the battle against the COVID-19 pandemic [5,22,23]. However, FFF produces components with poor surface quality compared to other AM processes. Due to the heating and cooling cycles involved in the FFF process, it has the inherent shortcomings of poor surface quality of produced items. The FFF process leads to a unique set of surface and dimensional defects, which appears to be a massive barrier to the functionality of FDM parts for rapid tooling and casting. The part configuration determines the surface finish of the manufactured component and the FFF process parameters selected [24,25].

Numerous researchers have enhanced the surface roughness of components fabricated by the FFF technique. The authors [26] testified that high build orientation and lower slice thickness lessen the surface roughness. A model for selecting the standard part alignment based on the surface roughness and manufacturing time of the component was established [27]. Ahn et al. found that the thickness of layers, surface angle, cross-sectional raster shape, and the overlap interval were significant parameters for surface roughness [28]. Bakar et al. reported that the contour width and layer thickness are significant parameters for the surface roughness [29]. Nancharaiah et al. concluded that the width of the raster and slice height influence the surface of parts [30]. Stephen Oluwashola Akande reported that small levels of print speed and line thickness result in improved surface roughness. However, there was no clear indication of the raster width if it was significant [31]. Nuñez et al. found that the lower layer thickness value with 100% density significantly impacts the surface roughness [32]. Chohan et al. investigated the FFF parameters and found layer thickness to be the significant factor [33]. Alsoufi et al. observed that the components of Acrylonitrile Butadiene Styrene (ABS) polymer have a higher surface roughness than other materials when the process conditions are the same as that of Polylactic Acid (PLA) [24]. According to Perez et al. [34], the decrease in thickness of deposited layers improves the surface quality of parts. The authors in their research [35,36] stated that the printing temperature and bed temperature should be chosen very carefully as a low bed temperature could detach the layer from the bed, and a high temperature could damage the layers. Similarly, the low printing temperature could clog the nozzle, and the high temperature could wrap up the material. Gao et al. found that the printing temperature, deposition speed, and layer thickness were important factors affecting the surface roughness [37]. Vyavahare et al. found that the printing temperature, deposition speed, and layer thickness influenced the surface roughness [38].

Taguchi optimization has become one of the best optimization methods and is a good tool for designing AM process parameters. The Taguchi method is an approach for system optimization that researchers use in various applications, including Computerized Numeric Control (CNC) operations [39,40], laser cutting [41,42], the EDM process [43], investment casting [44], and drilling [45]. The authors [46,47] proposed the Taguchi technique for process optimization—a quick, systematic way to improve operations quality, performance, and cost. Authors [48,49] used the Taguchi method to investigate the process parameters optimization of a FFF 3DP material. Srivastava et al. used the Taguchi and the grey relational analysis technique to analyze and optimize the FFF process parameters and obtained significant optimization [50]. In another research study, Vyavahare et al. studied the FFF process parameters, used the regression model to study the responses, and optimized the model [38].

After analyzing the literature, the processing parameters were selected that could affect the surface roughness or mechanical properties. The authors tried to take most of the parameters to understand the effect collectively. Print speed and raster width were selected to understand the effect on surface roughness, which are still unknown factors [51,52]. The literature shows that layer thickness is the key parameter in reducing surface roughness [30,53]. Build pattern greatly affects surface roughness, surface integrity, and mechanical properties [54].

Moreover, lightweight products are the main goal of industries, while reducing the weight leads to poor strength and low mechanical properties. Choosing the optimum infill pattern and infill percentage makes it possible to reach the highest strength while material consumption is lowest [55]. According to [52], the extrusion temperature still needs to be investigated for its effect on surface roughness. That is why the authors took this parameter. According to [35], bed temperature is the crucial factor affecting the 3DP part quality.

Much work has been performed on surface roughness optimization using different techniques from the literature. However, it is evident that: (i) work has rarely been performed to analyze the effect of more process parameters; (ii) no literature is available on the first layer thickness; (iii) there is insignificant work available on printing and optimization of standard Nylon-6 (PA-6) material. Thus, the objective of the study is as follows:

(i) To study the effect of FFF parameters (Initial Line Thickness (A), Raster Width (B), Bed Temperature (C), Build Pattern (D), Extrusion Temperature (E), Print Speed (F), and Layer Thickness (G)) and of ABS and PA-6 parameters (Layer Thickness (A1), Print Speed (A2) Extrusion Temperature (A3), and Build pattern (A4)) on the average surface roughness of ABS (Ra-ABS), the root-mean-square average surface roughness of ABS (Rq-ABS), the average surface roughness of PA-6 (Ra-PA-6), and the root-mean-square average surface roughness (Rq-PA-6);
(ii) To study the FFF 3DP parameters by the Taguchi Method using Analysis of variance (ANOVA) as well as the Signal to Noise (S/N) ratio for the confirmation test;
(iii) To develop a regression model to understand the parametric effect.

## 2. Materials and Methods

### 2.1. Materials

The experiment was carried out using an Ender 5S 3D printer with ABS and PA-6 material, 1.75 mm in diameter, and it was ready to print material. Specifications of ABS and PA-6 materials are given in Table 1, and they were bought from the “Yasin” company.

### 2.2. Methods

**Figure 2:**
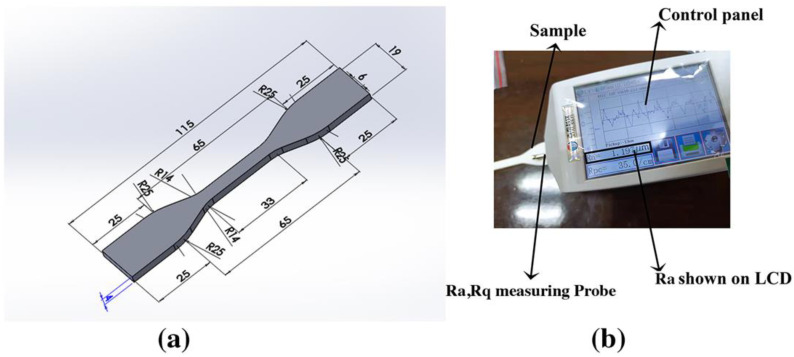
(**a**) STL D638 type iv sample with height 4 mm, length 115 mm, and width 19 mm; (**b**) JD 520 Ra tester.

The experimental sample was designed according to the D638 type IV sample because this design is feasible for the applications of the polymer material that are in line with the research [56], as shown in Figure 2a.

The range of FFF 3DP parameters for the current research is depicted in Table 2 and Table 3 for the ABS and PA-6 polymer, respectively. Taguchi L18 and L9, which are orthogonal array schemes, were applied for ABS and PA-6 to reduce experimental costs and efforts [35,57].

#### Measurement Procedure

The surface roughness tester from “JITAI KEYI” company, JD520 model, was used to obtain the surface roughness value of the FFF-fabricated part based on the ISO 16610–211 standard, as shown in Figure 2b, and Equation (1) describes the surface roughness value as the arithmetic means of absolute values of all deviations in the roughness profile measured along the complete length from the centerline. Equation (2) describes the Rq of the profile height deviations taken within the evaluation length and measured from the mean line [58]. Analytically, it was measured using a sampling length (*Lt*) = 4.8 mm and a cut-off wavelength of 0.8 mm following the ISO 16610–211 standard [59]. Specifications of the JD520 model surface roughness tester are depicted in Table 4.

A 100% density was taken in printing the samples because this value gives the minimum surface roughness [24,32,60]. The CAD prototype was made and converted into the STL format and then designed for the experiments.

(1) $$Ra=\frac{1}{Lt}\int_{0}^{Lt}\left|Z\left(x\right)\right|dx$$

(2) $$Rq={\left[\frac{1}{Lt}\int_{0}^{Lt}{\left(Z\left(x\right)\right)}^{2}dx\right]}^{\frac{1}{2}}$$

where *Lt* is the sampling length and *Z*(*x*) is the co-ordinate of the profile curve.

**Figure 3:**
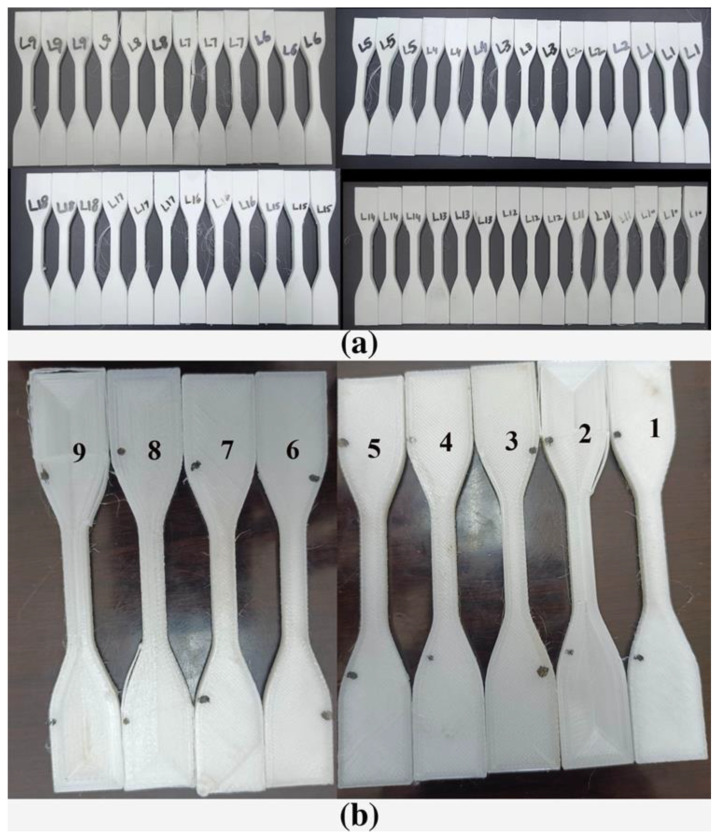
(**a**) FFF-Fabricated L18 experimental samples for ABS polymer. (**b**) FFF-Fabricated L9 experimental samples for PA-6 polymer.

After designing the experiment, the STL file was transferred to the slicer, the parameters were set, the model was sliced, and it was fabricated in a 3D printer. Then, the response was measured, an analysis was carried out using the Taguchi method, and a mathematical model was made to check the accuracy of the responses. Figure 3a shows the fabricated models of the ABS sample. The authors printed 3 samples for each test, measured six readings, and took the mean. The fabricated models PA-6 can be seen in Figure 3b. Due to the difficulty of printing, the authors printed one sample for each test, and then Ra-PA-6 and Rq-PA-6 were tested six times and taken as the mean to evaluate the RA-PA6.

## 3. Results and Discussions

### 3.1. Taguchi Process

Genichi Taguchi [61] employed this A loss function, which is the difference between the experimental and target values, which is then transformed into the S/N ratio. The S/N ratio is the ratio of the mean to the standard deviation. Dr. Taguchi adopted the term “signal” and “noise,” which refer to the desired response (mean) and unwanted (standard deviation) values. Taguchi classified the S/N ratio into three groups based on response requirements: higher-the-better, medium-the-better, and lower-the-better. In this investigation, the lower the quality parameters such as Ra and Rq, the better the surface quality. Equation (2) was used to compute the S/N ratio, with the findings displayed in Table 5 and Table 6 for ABS and PA-6, respectively. Minitab 20.0 was used to perform the Taguchi analysis.

(3) $$S/N\;\mathrm{ratio}\;\mathrm{for}\;\mathrm{the}\;\mathrm{smaller},\;\mathrm{the}\;\mathrm{better}=-10\;\log\frac{1}{N}\sum {\left(R\right)}^{2}$$

where:

*N* = Number of observations;
*R* = Observed value for each response.

L18 parameters, responses, and the S/N ratio findings for the ABS polymer from Equation (3) are depicted in Table 5.

**Table 5:** Experimental design, results, and their computed S/N ratios for ABS polymer.

A (mm)	B (mm)	C (°C)	D	E (°C)	F (mm/s)	G (mm)	Ra (µm)	Rq (µm)	SNRa	SNRq
0.2	0.3	85	1	225	50	0.08	2.437	2.924	−7.737	−9.320
0.2	0.3	90	2	230	60	0.16	3.004	3.694	−9.554	−11.352
0.2	0.3	95	3	235	70	0.24	3.605	4.398	−11.138	−12.865
0.2	0.4	85	1	230	60	0.24	3.788	4.924	−11.568	−13.847
0.2	0.4	90	2	235	70	0.08	2.807	3.480	−8.964	−10.833
0.2	0.4	95	3	225	50	0.16	3.215	3.922	−10.143	−11.870
0.2	0.5	85	2	225	70	0.16	4.112	5.098	−12.281	−14.149
0.2	0.5	90	3	230	50	0.24	3.933	4.719	−11.894	−13.478
0.2	0.5	95	1	235	60	0.08	2.474	3.043	−7.867	−9.666
0.3	0.3	85	3	235	60	0.16	3.012	3.795	−9.577	−11.584
0.3	0.3	90	1	225	70	0.24	3.709	4.599	−11.385	−13.253
0.3	0.3	95	2	230	50	0.08	2.099	2.581	−6.440	−8.238
0.3	0.4	85	2	235	50	0.24	3.399	4.282	−10.627	−12.634
0.3	0.4	90	3	225	60	0.08	2.914	3.613	−9.289	−11.158
0.3	0.4	95	1	230	70	0.16	3.217	3.924	−10.149	−11.876
0.3	0.5	85	3	230	70	0.08	3.359	4.165	−10.524	−12.392
0.3	0.5	90	1	235	50	0.16	2.8975	3.621	−9.240	−11.178
0.3	0.5	95	2	225	60	0.24	4.045	5.258	−12.138	−14.417

L9 parameters, responses, and the S/N ratio findings from the PA-6 polymer are depicted in Table 6.

**Table 6:** Experimental design, results, and their computed S/N ratios for PA-6 polymer.

A1 (mm)	A2 (mm/s)	A3 (°C)	A4	Ra (μm)	Rq (μm)	SNRA1	SNRA2
0.1	40	250	1	21.469	26.421	-26.521	−28.439
0.1	50	255	2	21.675	26.61	−26.6362	−28.5009
0.1	60	260	3	21.766	26.772	−26.7192	−28.5536
0.2	40	255	3	22.184	27.251	−26.7556	−28.7076
0.2	50	260	1	22.188	27.313	−26.9208	−28.7274
0.2	60	250	2	22.393	27.543	−26.9224	−28.8002
0.3	40	260	2	22.554	27.518	−27.0022	−28.7923
0.3	50	250	3	22.965	28.176	−27.0645	−28.9976
0.3	60	255	1	22.855	27.883	−27.2213	−28.9068

### 3.2. Effects of the FFF Parameters on Surface Roughness

#### 3.2.1. Effects of the FFF Parameters on Ra-ABS and Rq-ABS

The Parameter “A” is rarely studied. Thus, the authors took the standard values by the Cura software of one maximum value to understand the effect on surface roughness. A high “A” made it stable and could be printed well. For parameter “B”, according to the literature, the significance is unknown, so the raster width is rarely studied [52]. Thus, the authors took the Cura software’s standard values and took lower, standard, and higher values to understand the parametric trend. A value lower than 0.3 took too much time to print. Thus, the authors printed 0.3 as the lowest level, 0.4 as the standard or middle level, and 0.5 as the highest level. The success rate of printing the first layer during AM is significantly related to the parameter “C” [35]. Initially, four different bed temperatures, i.e., 83 °C, 87 °C, 93 °C, and 98 °C, were used for testing. The molten ABS material could not be attached to the bed base when the bed temperature was set to 83 °C, due to low bed temperature. Conversely, the solidification time of the molten ABS material increased when the bed temperature was set to 98 °C. Thus, the authors took 85 °C as the lowest value for “C” and 95 °C as the highest value. Parameter “D” plays a vital role in part strength and the surface finish. A broad range of patterns such as grid, triangle, zigzag (3), and concentric (2) were generated and produced by FDM printers [54], where pattern 2 showed improved results. Another study [62] reported that a linear pattern (1) is better than a triangle and tetrahedral pattern. Thus, there was a need to evaluate between pattern “1” and pattern “2”. Thus, the authors took 3 patterns “1”, “2”, and “3” to understand the parametric trend for surface roughness.

The Parameter “E” was recommended to be set around 220–250 °C by the “Yasin” company. Therefore, five different nozzle temperatures, i.e., 220 °C, 225 °C, 230 °C, 235 °C, and 240 °C, were used for testing. The ABS filament could not be melted when the nozzle temperature was set to 220 °C. Conversely, the burning of ABS filaments was observed when the nozzle temperature was set to 240 °C. Thus, the authors took 225 °C as the lowest value for “E” and 235 °C as the highest value. For parameter “F”, the authors tried to print less than 50 mm/s, and due to the effect of other parameters, the samples took too long; some took more than 12 h for 3 strips that were not efficient. Thus, the authors took 50 mm/s as the lowest speed and the highest as 70. The line thickness was mostly taken at 0.1–0.3, and 0.1 mm significantly reduced the surface roughness [63]. The authors tried to print at 0.08 mm as a lower value and succeeded in ABS, while they could not print lower than 0.1 mm in PA6. Thus, the authors took 0.1 as the lowest parameter for PA6 and 0.08 for ABS.

**Figure 4:**
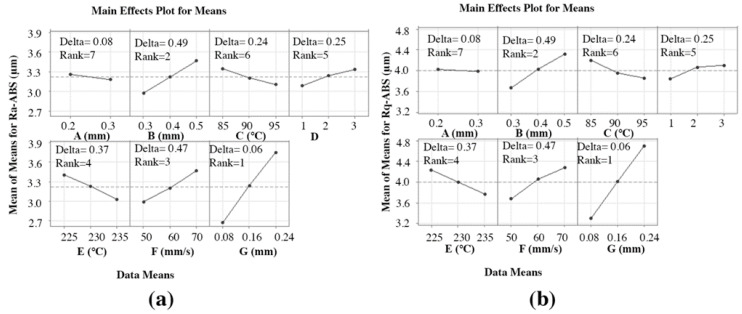
Effect of FFF parameters on polymers: (**a**) Ra-ABS and (**b**) Rq-ABS.

Figure 4 represents the effect of FFF parameters on Ra-ABS and Rq-ABS. Ra-ABS and Rq-ABS decreased with an increase in “A”. Increased “A” led to a better attachment of the sample on the bed while printing, which causes fewer vibrations during the 3DP process. However, it did not play a significant role in decreasing the surface, as it lay in rank 7 with a delta value of 0.08. Ra-ABS and Rq-ABS were significantly influenced by “B” as it lay in rank 2 with delta values of 0.492 and 0.65, respectively. It is evident from Figure 4 that the Ra-ABS and Rq-ABS values increased as “B” increased. This is because the higher value of “B” will expand the perimeter of beads, which will need a stylus to travel longer distances, leading to poor Ra-ABS and Rq-ABS [64]. The optimal value of “C” is needed to increase the quality of the 3D-printed part. A low value of “C” could cause the material to detach from the bed, while a high temperature could distort the material and lead to solidification. It is observed that the Ra-ABS and Rq-ABS decreased by increasing “C” from 85 °C to 95 °C because it helped the layers to attach to the bed firmly [35]. However, it did not impact significantly, as “C” for Ra-ABS lay in rank 6 with a delta value of 0.242 and “C” for Rq-ABS lay in rank 5 with a delta value of 0.35. The parameter “D” for Ra-ABS ranked 5 with a delta value of 0.253, while “D” for Rq-ABS ranked 6 with a delta value of 0.26. It gave the optimal value to determine the least Ra-ABS and Rq-ABS as factor “3” of “D” caused an increment in Ra-ABS and Rq-ABS as pattern “3” due to the triangular structures, which are nonoptimal and have poor adhesion with the bed.

In contrast, the “1” gave the least Ra-ABS and Rq-ABS because the best adhesion in layers was joined together in lines [54]. Ra-ABS and Rq-ABS decreased with increasing “E” because the high temperature increases the molten flow, and it helps to decrease the surface roughness [65]. The parameter “E” ranked 4 with a delta value of 0.373 and 0.46 for Ra-ABS and Rq-ABS respectively, which shows that it is a vital parameter affecting the Ra-ABS and Rq-ABS. Ra-ABS and Rq-ABS increased significantly with increasing “F” factor because the high speed could create ringing or ghosting artifacts or even layer shifting [50]. The parameter “F” ranked 3 with a delta value of 0.474 and 0.6 for Ra-ABS and Rq-ABS respectively, which proves the importance of “F” in printing quality. The layer-by-layer procedure produces the staircase effect, leading to increased Ra-ABS and Rq-ABS [7]. The staircase effect can be significantly reduced when the manufacturing parameters, especially “G”, are properly selected. Thus, a lower “G” significantly decreased the Ra-ABS and Rq-ABS. It is also evident that it lay in rank 1 with delta values of 1.065 and 1.39 for Ra-ABS and Rq-ABS respectively, showing the most significant parameter in improving the Ra-ABS and Rq-ABS polymer.

The obtained trend for Ra-ABS and Rq-ABS for increasing “A”, “C”, and “E”, while decreasing “D”, “F”, and “G”, respectively, agrees with the results of the research literature during parametric optimization for Ra-ABS and Rq-ABS using the FFF technique [7,35,50,54,64,65,66,67]. The factors “G” and “F” played a significant role on Ra-ABS and Rq-ABS and found significantly less values for Ra-ABS and Rq-ABS.

**Figure 5:**
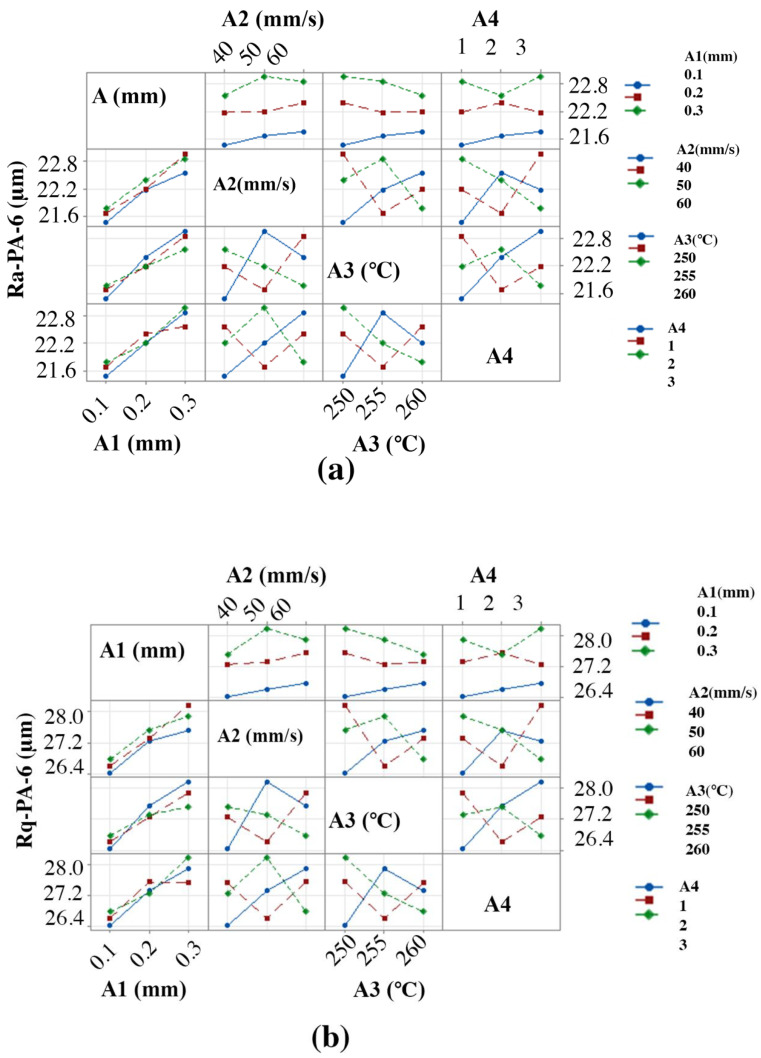
Effect of parametric interactions on polymers: (**a**) Ra-ABS and (**b**) Rq-ABS.

Parametric interactions were assessed to understand the interaction effect on Ra-ABS and Rq-ABS. The interaction plot of Figure 5 indicates that interactions are present for all factors except “A”. There is a significant effect of interaction between “G” and “B”, where small raster and small thickness help significantly to reduce the Ra-ABS and Rq-ABS, and the interaction between “F” and “E”, which shows that the high “E” melted the material well, while the low “F” spread out the layer without distortion, resulting in an improved Ra-ABS and Rq-ABS.

**Figure 6:**
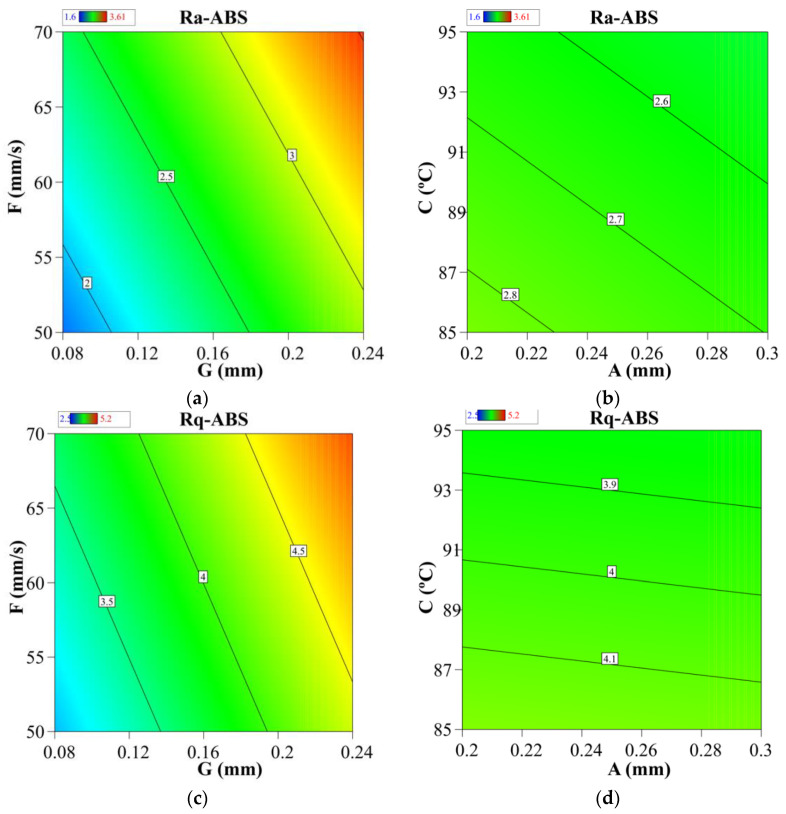
Contour graphs: (**a**) “G” vs. “F” for Ra-ABS, (**b**) “A” vs. “C” for Ra-ABS, (**c**) “G” vs. “F” for Rq-ABS, and (**d**) “A” vs. “C” for Rq-ABS.

Contour plots demonstrate the relationship between two control variables and a response variable by displaying the expected response variables’ discrete contours. The contour plots in Figure 6 illustrate the relationship between the process parameters and the Ra-ABS value. The relationships of two most significant and two least significant factors were analyzed. According to Figure 6a, a low level of “G” and a low level of “F” resulted in the development of a significant low Ra-ABS value. As seen in Figure 6b, the low Ra-ABS was achieved at high “C” and high value “A” values, respectively. However, the “C” and “A” values did not significantly affect the surface roughness. As seen in Figure 6c, a low level of “G” and a low level of “F” resulted in the development of a low Rq-ABS value. As seen in Figure 6d, the low Rq-ABS may be achieved at high “C” and high value “A” values, respectively. Section 3.1 discusses the scientific basis for the preceding observation. The results agree with the research literature during the optimization of FFF parameters [35,61].

#### 3.2.2. Effects of the FFF Parameters on Ra-PA-6 and Rq-PA-6

**Figure 7:**
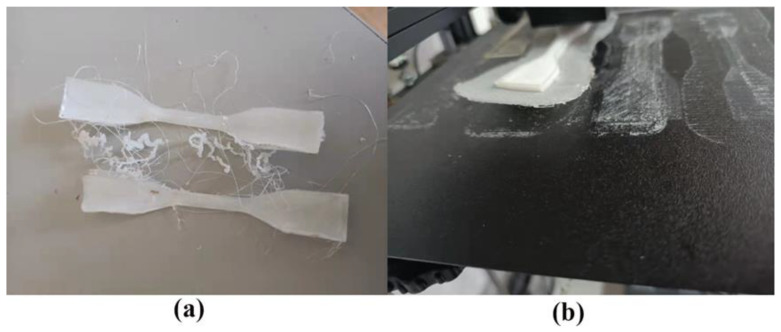
(**a**) Failed print while taking 7 parameters; (**b**) wrapped print at 245 °C.

PA-6 is rarely successfully printed because of its property of wrapping up, the heat effect, and quick solidification [37]. Authors tried to print PA-6 with the Creality CR5 3D Printer with 7 parameters (A, B, C, D, E, F, and G) that were taken for ABS as well in Section 2 but ended up with no print because the probability of changing the parameter’s value could not be high enough to succeed in printing, as shown in Figure 7a. The authors tried to print using 4 parameters using A1, A2, A3, and A4 with Creality CR5 and achieved success. The authors tried to print at a 245 °C print temperature, and the print wrapped up and could not attach to the bed surface, as depicted in Figure 7b. Thus, the authors took 250 °C as the lowest parameter of PA-6 and 260 as the highest parameter. The authors could not print at 0.08 mm A1. Thus, 0.1 mm was taken as the lowest and 0.3 as the highest parameter. The authors tried to print the material at 40 mm/s, 50 mm/s, 60 mm/s, 70 mm/s, and 80 mm/s as the lowest and highest parametric values are recommended by the material manufacturer. However, the material could not be printed above 60 mm/s. Thus, 40 mm/s was taken as level 1, 50 mm/s as level 2, and 60 mm/s as level 3. However, there was a negligible change in Ra-PA-6 and Rq-PA-6 because of the open-air printer as the air quickly wraps up and forms defects in layers [68].

**Figure 8:**
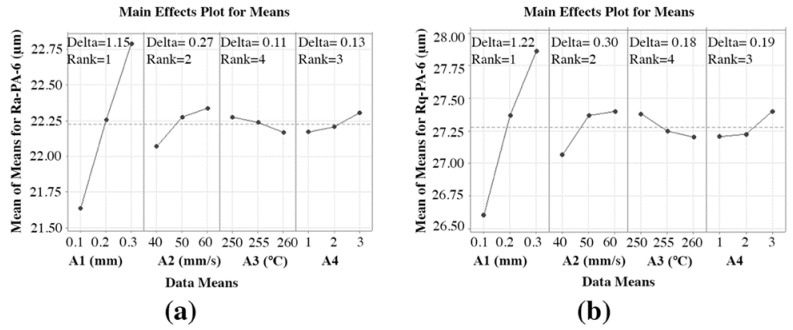
Effect of FFF parameters on polymers: (**a**) Ra-PA-6 and (**b**) Rq-PA-6.

Figure 8a,b depict the effect of the FFF parameters on Ra-PA-6 and Rq-PA-6, respectively. The sample was printed in the open-air printer. Ra-PA-6 and Rq-PA-6 decreased significantly when “A1” was decreased because the staircase effect was limited and the surface was smooth. It is also evident that it lay in rank 1 with delta values of 1.15 and 1.22 for Ra-PA-6 and Rq-PA-6, respectively, showing the most significant parameter in improving the Ra-PA-6 and Rq-PA-6 polymer. Ra-PA-6 and Rq-PA-6 decreased significantly by decreasing the “A2” from 60 mm/s to 40 mm/s. It gave a smoother surface. The parameter “A2” ranked 2 with a delta value of 0.27, which proves the importance of “A2” in printing quality. Ra-PA-6 and Rq-PA-6 decreased by increasing A3 because increasing “A3” melted well and helped the material to lie down smoothly before it wrapped up. The parameter “A3” ranked 4 with a delta value of 0.11, which shows that they were not the significant factors in improving the surface roughness. Ra-PA-6 and Rq-PA-6 decreased in pattern 1, while more surface roughness was observed in pattern 3 because of the uneven pattern. The parameter “A4” ranked 3 with a delta value of 0.13. The findings match the available general literature such as [37,69,70,71,72].

**Figure 9:**
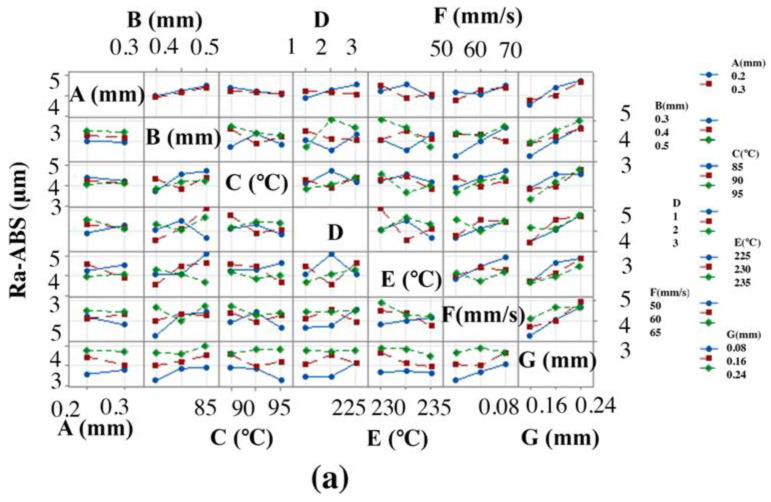

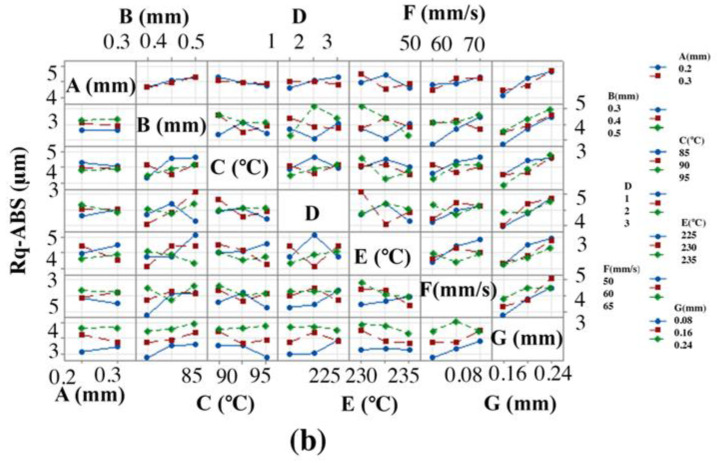
Effect of parametric interactions on polymers: (**a**) Ra-PA-6 and (**b**) Rq-PA-6.

Parametric interactions were assessed to understand the interaction effect on Ra-PA-6 and Rq-PA-6. The interaction plot in Figure 9a,b indicates severe interactions present for all parameters. There is a significant effect of interaction between “A” and “A3” and “A2”, where high temperature melts the material, the smallest level of “A” creates thinner lines, and smallest level of “A2” creates a minimized stair-case effect, which eventually helps significantly to reduce the Ra-PA-6 and Rq-PA-6 [73].

**Figure 10:**
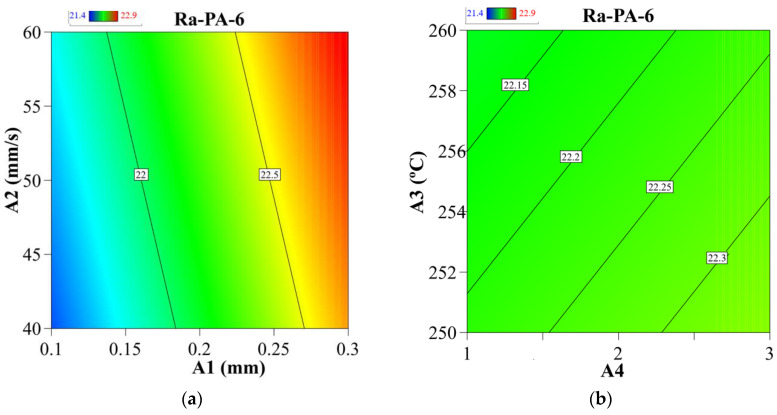

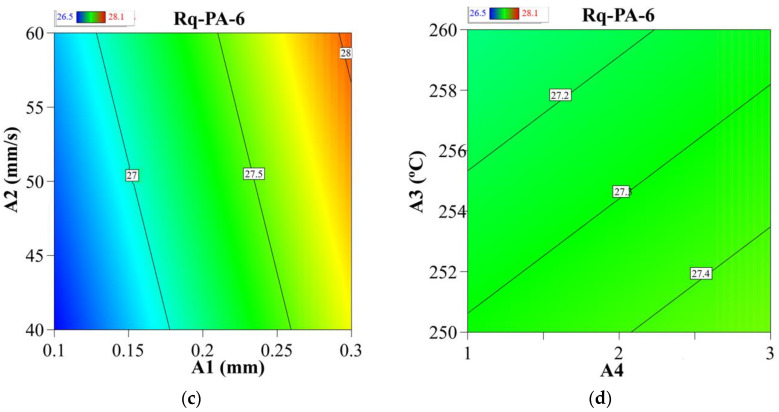
Contour graphs: (**a**) “A2” vs. “A1” for Ra-PA-6, (**b**) “A4” vs. “A3” for Ra-PA-6, (**c**) “A2” vs. “A1” for Rq-PA-6, and (**d**) “A4” vs. “A3” for Rq-PA-6.

Figure 10 illustrates contour graphs illustrating the relationship between FFF parameters and Ra-PA-6 polymer. The contour plots in Figure 10 illustrate the relationship between the process parameters and the Ra-PA-6 and Rq-PA-6 values. The relationship of two most significant and two least significant factors was analyzed. According to Figure 10a, a low level of “A1” and a low level of “A2” resulted in the significant decrease in Ra-ABS value. As seen in Figure 10b, a low Ra-PA-6 was achieved at high “A3” and low “A4” values. However, “A3” and “A4” did not significantly affect the surface roughness. As seen in Figure 10c, a low level of “A1” and a low level of “A2” resulted in the significant decrease in Rq-PA-6 value. As seen in Figure 10d, the low Rq-PA-6 was achieved at high “A3” and low “A4” values, respectively. Section 3.1 and Section 3.2 discuss the scientific basis for the preceding observation. The results agree with the research literature during the optimization of FFF parameters [73,74].

### 3.3. ANOVA for Ra-ABS, Rq-ABS, Ra-PA-6, and Rq-PA-6

The ANOVA pinpoints the FFF parameter that has the maximum impact on performance. Table 7 and Table 8 show the ANOVA analysis results for Ra-ABS and Rq-ABS and Ra-PA-6 and Rq-PA-6, respectively. Table 7 shows that “G” has the greatest influence on Ra-ABS and Rq-ABS, followed by “F”, “B”, “E”, “D”, “C”, and “A”, in that order. The percentage contribution of “G”, “F”, “B”, “E”, “D”, “C”, and “A” on Ra-ABS was 60.38%, 13.01%, 12.49%, 5.78%, 4.01%, 3.7%, and 0.4%, respectively.

The percentage contribution of “G”, “F”, “B”, “E”, “D”, “C”, and “A” on Rq-ABS was 60.01%, 13.17%, 12.97%, 5.00%, 4.26%, 3.05%, and 0.04%, respectively. From the ANOVA analysis, it is evident that surface roughness was influenced significantly by “G”, “A1”, “F”, and “A2”.

Ra-PA-6 and Rq-PA-6 were mostly influenced by A1, followed by “A4”, “A2”, and “A3”. The percentage contribution of “A1”, “A2”, “A4”, and “A3” was 92.48%, 5.49%, 1.35%, and 0.72%, respectively, as depicted in Table 9.

For Rq-PA-6, the percentage contribution of “A1”, “A2”, “A4”, and “A3” was 92.45%, 5.48%, 1.34%, and 0.71%, respectively, as depicted in Table 10.

### 3.4. The Selection of Optimal Parametric Conditions for Ra-ABS, Rq-ABS, Ra-PA-6, and Rq-PA-6

**Figure 11:**
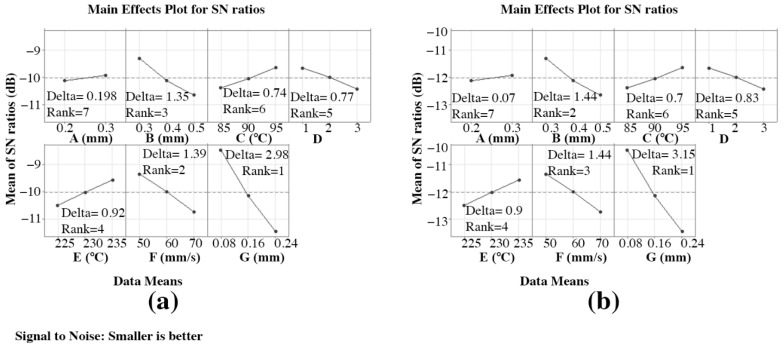
Mean S/N ratio for polymers: (**a**) Ra-ABS and (**b**) Rq-ABS.

**Figure 12:**
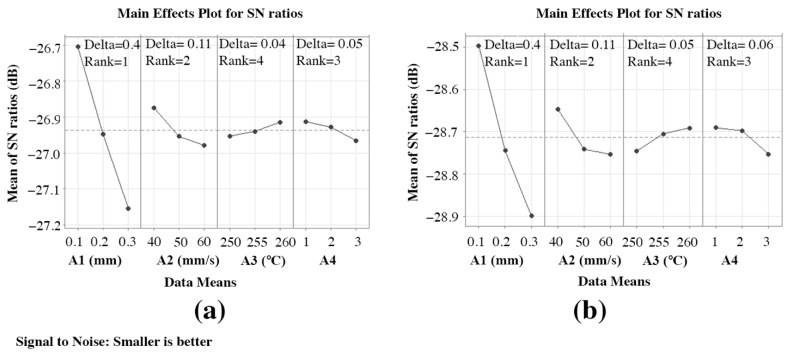
Mean S/N ratio for polymers: (**a**) Ra-PA-6 and (**b**) Rq-PA-6.

The achieved S/N ratio response tables for Ra-ABS, Rq-ABS, Ra-PA-6, and Rq-PA-6 are shown in Table A1, Table A2, Table A3 and Table A4, respectively. Figure 11 and Figure 12 reflect the ABS and PA-6 mean S/N ratio graphs computed in Minitab, respectively. A high S/N ratio indicates that the divergence between the desired and measured outputs is as small as possible [56]. From Figure 11a,b, the highest mean S/N ratio achieved for Ra-ABS and Rq-ABS are “A” at 0.3 mm, “B” at 0.3 mm, “C” at 95 °C, D at “1”, “E” at 235 °C, “F” at 50 mm/s, and “G” at 0.08 mm. Hence, the predicted optimal FFF parameters for achieving the low Ra-ABS and Rq-ABS using the Taguchi technique were found as A = 0.3 mm, B = 0.3 mm, C = 95 °C, D = 1, E = 235 °C, F = 50 mm/s, and G = 0.08 mm, and the corresponding values were bolded in Table A1 and Table A2 to make the response table easier to interpret. This predicted optimal combination was symbolized as “A-S2 B-S1 C-S3 D-S1 E-S3 F-S1 G-S1” for Ra-ABS and Rq-ABS. The S/N ratio achieved for Ra-ABS and Rq-ABS influencing “A” lies in rank 7 with delta values of 0.198 and 0.07, respectively; “B” lies in rank 3 and 2 with delta values of 1.352 and 1.44, respectively; “C” lies in rank 6 with delta values of 0.74 and 0.7, respectively; D lies in rank 5 with delta values of 0.77 and 1.83, respectively; “E” lies in rank 4 with delta values of 0.927 and 0.9, respectively; “F” lies in rank 2 and 3 with delta values of 1.392 and 1.44, respectively; “G” lies in rank 1 with delta values of 2.988 and 3.15, respectively, which shows the significance of each parameter, especially “G”, “F”, and “B”.

From Figure 12a,b, the highest mean S/N ratio achieved for Ra-PA-6 and Rq-PA-6 are “A1” at 0.1 mm, “A2” at 40 mm/s, “A3” at 260, and “A4” at 1, respectively. Table A3 and Table A4 show the achieved S/N ratio response table for Ra-PA-6 and Rq-PA-6. The means of the S/N ratio for the PA-6 are denoted in the graph shown in Figure 12. From Figure 12, the estimated optimal FFF parameters for achieving low Ra-PA-6 and Rq-PA-6 were found to be A1 = 0.1 mm, A2 = 40 mm/s, A3 = 260, and A4 = 1. This predicted optimal combination was symbolized as “A1-S1 A2-S1 A3-S3 A4-S1” for Ra-PA-6 and Rq-PA-6. The S/N ratio achieved for Ra-ABS and Rq-ABS influencing “A1” lies in rank 1 with a delta value of 0.45, “A2” lies in rank 2 with a delta value of 0.11, “A3” lies in rank 4 with a delta value of 0.04, and A4 lies in rank 3 with a delta value of 0.05, which shows the significance of each parameter, especially “A1” and “A2”.

### 3.5. Validation Test

Confirmation experiments are required to validate Taguchi’s predicted optimal conditions. The predicted S/N ratio ($\varepsilon_{predicted}$) was determined using Equation (4) [75] to estimate and evaluate the responses at predicted optimal Ra conditions.

(4) $$\varepsilon_{predicted}=\varepsilon_{l}+\overset{x}{\underset{i=0}{\sum }}\varepsilon_{0}-\varepsilon_{l}$$

$\varepsilon_{l}$ = Total mean of S/N ratio;
$\varepsilon_{0}$ = Mean S/N ratio at optimum level;
*x* = Number of the input FFF parameters.

The confirmation experiments were carried out at the Taguchi predicted optimal printing settings, and the results are reported in Table A5 and Table A6 for Ra-ABS, Rq-ABS, Ra-PA-6, and Rq-PA-6, respectively. Roughness performance characteristics improve when the predicted optimum printing circumstances are used. Table A5 and Table A6 show that the S/N ratios of the predicted and optimum printing conditions are fairly close for both polymers. Compared to preliminary parameter values, the S/N ratio improvements for Ra-ABS, Rq-ABS, Ra-PA-6, and Rq-PA-6 under the ideal FFF printing condition were 4.474 dB, 4.473 dB, 0.36 dB, and 1.18 dB, respectively. According to the confirmation experiments, the Taguchi predicted optimal printing circumstances produce better outcomes than the initial parameter conditions. When comparing initial parameter circumstances to Taguchi’s projected optimal printing conditions, Ra-ABS, Rq-ABS, Ra-PA-6, and Rq-PA-6 reductions were 85.9%, 4.87%, 96.7%, and 4.33%, respectively. As a result, the Taguchi predicted optimal printing circumstances for attaining low surface roughness in the FFF 3DP technique under the given circumstances were used as the optimal printing conditions.

**Figure 13:**
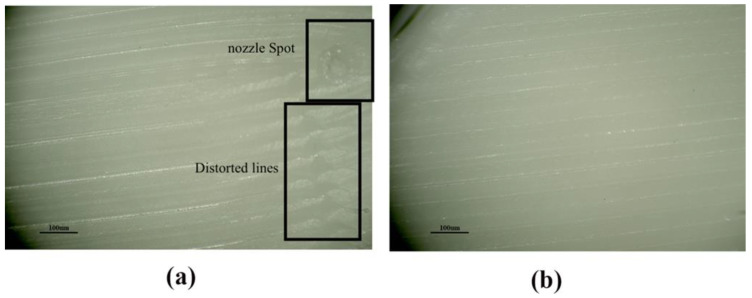
Surface roughness Tester showing the Ra-ABS and Rq-ABS at (**a**) Initial parameters setting at A = 0.3 mm, B = 0.4 mm, C = 100 °C, D = 2, E = 230 °C, F = 60 mm/s, and G = 0.16 mm; (**b**) Taguchi optimal settings at A = 0.3 mm, B = 0.3 mm, C = 95 °C, D = 1, E = 235 °C, F = 50 mm/s, and G = 0.08 mm.

**Figure 14:**
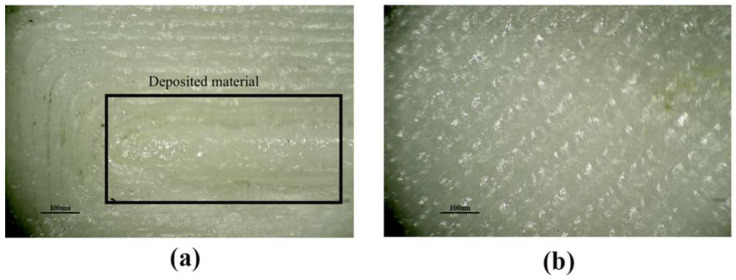
Surface roughness Tester showing the Ra-ABS and Rq-ABS at (**a**) Initial parameters setting at A1= 0.2 mm, A2= 50 mm/s, A3= 255 °C, and A4 = 2; (**b**) Taguchi optimal settings at A1= 0.1 mm, A2 = 40 mm/s, A3 = 260 °C, and A4 = 1.

For surface roughness, Figure 13a,b and Figure 14a,b show microscopic images produced from the XTZ microscope at 20× under initial parameter circumstances and Taguchi optimum printing conditions, respectively. Figure 13a has spots due to concentric movement, and lines are distorted, while Figure 13b is spotless.

Figure 14a shows a lot of PA-6 material placed in the middle of lines, creating more roughness, while Figure 14b has clear lines. The Taguchi optimal printing circumstances resulted in low Ra-PA-6 and Rq-PA-6 values.

## 4. Mathematical Modeling

The predictive models for the response variable of surface roughness as a function of “A”, “B”, “C”, “D”, “E”, “F”, and “G” and “A1”, “A2”, “A3”, and “A4” were developed using the regression analysis in Minitab 20.0. On each response, no modification was applied. Equations (5)–(8) provide the prediction equations generated from the regression analysis for Ra-ABS, Rq-ABS, Ra-PA-6, and Rq-PA-6.

Ra-ABS = 10.463 − 0.804 A (mm) + 2.462 B (mm) − 0.02420 C (°C) + 0.1263 D − 0.03729 E (°C) + 0.02357 F (mm/s) + 6.655 G (mm)(5)

Rq-ABS (µm) = 13.14 − 0.404 A (mm) + 3.261 B (mm) − 0.03437 C (°C) + 0.1313 D − 0.04658 E (°C) + 0.03012 F (mm/s) + 8.723 G (mm)(6)

Ra-PA-6 (μm) = 22.98 + 5.773 A1 (mm) + 0.01345 A2 (mm/s) + 0.01063 A3 (°C) + 0.0672 A4(7)

Rq-PA-6 (μm) = 29.55 + 6.290 A1 (mm) + 0.01680 A2 (mm/s) − 0.0179 A3 (°C) + 0.0970 A4 (8)

**Figure 15:**
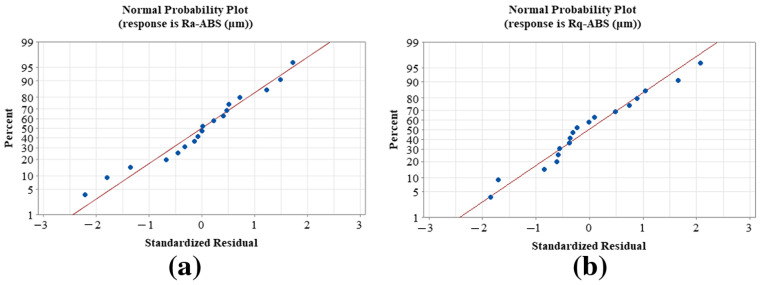
Normal probability graph for the residuals of polymers: (**a**) Ra-ABS and (**b**) Rq-ABS.

The created models’ competence was evaluated using R^2^, which is the coefficient of determination [76]. The coefficient of determination has a value between zero and one. If it is near one, it implies that the independent and dependent variables are well-matched [77]. If R^2^ = 94%, it signifies that the new data were assessed with a variability of 94%. In this work, the generated mathematical models for Ra-ABS and Rq-ABS had high R^2^ values of 99.72% and 98.5%, respectively. The generated mathematical models for Ra-PA-6 and Rq-PA-6 had high R^2^ values of 99.2% and 98.4%, respectively. The residual graphs were used to determine the relevance of the anticipated model’s coefficients [78]. If the residual graph is straight, the model’s residual errors are regularly spread and the coefficients are significant [79]. Figure 15 shows the residual plots for Ra-ABS and Rq-ABS, where the residuals for Ra-ABS and Rq-ABS are close to the straight line, indicating that the created model coefficient models are significant.

**Figure 16:**
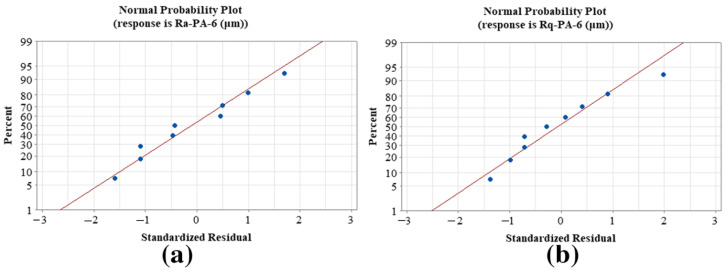
Normal probability graph for the residuals of polymers: (**a**) Ra-PA-6 and (**b**) Rq-PA-6.

Figure 16 shows the residual plots for Ra-PA-6 and Rq-PA-6, where the residuals Ra-PA-6 and Rq-PA-6 are close to the straight line, indicating that the created model coefficient models are significant.

Conformance tests were performed to verify the constructed models; the results are presented in Table 11. The testing findings were randomly selected from the L9 and L18 orthogonal experimental designs. According to the confirmation findings, it was discovered that the anticipated values from the models and the experimental values were in excellent agreement within the provided parameter range.

## 5. Conclusions and Prospects

The following decisions were made from the results of the experimental investigation:

- The lowest average surface roughness for Acrylonitrile Butadiene Styrene (Ra-ABS) and the root-mean-square average surface roughness for Acrylonitrile Butadiene Styrene (Rq-ABS) were found at high initial line thickness, high raster width, high bed temperature, high line build pattern, high extrusion temperature, low print speed, and low level of layer thickness.
- The Taguchi technique helped to reduce Ra-ABS by 85.9% and Rq-ABS by 96.7% under optimal printing conditions.
- The lowest average surface roughness for Nylon-6 (Ra-PA-6) and root-mean-square average surface roughness for nylon-6 (Rq-PA-6) were found at low layer thickness (A1), low print speed (A2), high extrusion temperature (A3), and high line build pattern (A4).
- Taguchi determined that optimal printing conditions reduced Ra-PA-6 and Rq-PA-6 by 4.8% and 4.33%, respectively, because PA-6 is hard to print in an open-air printer as it absorbs moisture.
- From the Analysis of Variance (ANOVA), Ra-ABS, Rq-ABS, Ra-PA-6, and Rq-PA-6 were significantly influenced by the “G”, “F”, “A1”, and “A2.
- It was seen from the results that the Taguchi-determined optimal printing conditions lessened the surface roughness during the Fused Filament Fabrication (FFF) approach. Hence, it was recommended that polymer printing industries use such optimal printing conditions to improve the printing quality of ABS and PA-6 polymers within these given ranges.
- The predicted response findings and experimental results were close using the created mathematical models for surface roughness. As a result, the generated models might be utilized to determine the best printing conditions for evaluating product quality without trial tests requiring much time to print materials.

### Future Recommendations

- Use different kinds of PA-6, which could give less Ra and Rq.
- More PA-6 parameters should be investigated, and practical industrial models should be fabricated using these values.
- Perform tensile and flexural tests to find the mechanical properties of PA-6.
- Consider reducing the printing time and making it more economical.
- Use different optimizing techniques such as the response surface methodology to improve the surface roughness further.

## Figures and Tables

**Table 1:** Specifications of materials for experiments (Data credit: Yasin).

TYPE	Filament Diameter	Print Speed mm/s	Printing Temperature °C	Bed Temperature °C	Tensile Strength MPa	Bending Strength MPa
ABS	1.75 mm	60–100	220–250 °C	80–120 °C	47	76
PA6	1.75 mm	40–80	220–285 °C	80–100 °C	65	85

**Table 2:** FFF 3DP parameters used to print ABS polymer sample.

Parameter	Unit	Symbol	Level 1	Level 2	Level 3
Initial Line Thickness	mm	A	0.2	0.3	
Raster Width	mm	B	0.3	0.4	0.5
Bed Temperature	°C	C	85	90	95
Build Pattern		D	Line (1)	Concentric (2)	Zigzag (3)
Extrusion Temperature	°C	E	225	230	235
Print Speed	mm/s	F	50	60	70
Line Thickness	mm	G	0.08	0.16	0.24

**Table 3:** FFF 3DP parameters used to print ABS polymer sample.

Parameter	Unit	Symbol	Level 1	Level 2	Level 3
Line Thickness	mm	A1	0.1	0.2	0.3
Print Speed	mm/s	A2	40	50	60
Extrusion Temperature	°C	A3	250	255	260
Build Pattern		A4	Line (1)	Concentric (2)	Zigzag (3)

**Table 4:** Specifications of JD520 tester.

Measuring Surface Roughness		Resolving Power	Measurement Items	Sampling Length (mm)	Evaluation Length	Indication Error	Precision
*Z*-Axis	*X*-Axis	*Z*-Axis Vertical					
320 µm	17.5 mm	0.01 µm/±20 µm 0.02 µm/±40 µm 0.04 µm/±80 µm 0.08 µm/±160 µm	Ra, Rz, Rq, Rt, Rp, Rv, R3z, R3y, Rz(JIS), Rs, Rsk, Rsm, Rku, Rmr, Ry(JIS), Rmax, RPc, Rk, Rpk, RVk, Mr1, Mr2	0.25, 0.8, 2.5	Ln = lr × *n*, *n* = 1 − 5	Not more than 10%	0.001 µm

**Table 7:** ANOVA Table for the Ra-ABS polymer.

Source	DF	Seq SS	Adj SS	Percentage Contribution
A (mm)	1	0.1756	0.1756	0.4
B (mm)	2	5.5685	2.7843	12.49
C (°C)	2	1.6468	0.8234	3.7
D	2	1.7853	0.8926	4.01
E (°C)	2	2.5762	1.2881	5.78
F (mm/s)	2	5.8313	2.9156	13.01
G (mm)	2	26.9307	13.4653	60.38
Residual Error	4	0.0879	0.022	0.20
Total	17	44.6023		100

**Table 8:** ANOVA Table for the Rq-ABS polymer.

Source	DF	Seq SS	Adj SS	Percentage Contribution
A (mm)	1	0.0234	0.0234	0.04
B (mm)	2	6.4404	6.4404	13.17
C (°C)	2	2.0825	2.0825	4.26
D	2	1.4910	1.4910	3.05
E (°C)	2	2.4458	2.4458	5.00
F (mm/s)	2	6.3409	6.3409	12.97
G (mm)	2	29.8269	29.8269	61.01
Residual Error	4	0.2330	0.2330	0.47
Total	17	48.8840		100

**Table 9:** ANOVA Table for the Ra-PA-6 polymer.

Source	DF	Seq SS	Adj SS	Percentage Contribution
A1 (mm)	2	0.306476	0.306476	92.48
A2 (mm/s)	2	0.018182	0.018182	5.49
A3 (°C)	2	0.002373	0.002373	0.72
A4	2	0.004467	0.004467	1.35
Total	8	0.331498		100

**Table 10:** ANOVA Table for the Rq-PA-6 polymer.

Source	DF	Seq SS	Adj SS	Percentage Contribution
A1 (mm)	2	0.245653	0.245653	92.45
A2 (mm/s)	2	0.020776	0.020776	5.48
A3 (°C)	2	0.004804	0.004804	0.71
A4	2	0.006916	0.006916	1.34
Total	8	0.278149		100

**Table 11:** Confirmation model for the developed mathematical model.

Run		Experimental			Predicted				Error%			
	Ra-ABS (µm)	Rq-ABS (µm)	Ra-PA-6 (µm)	Rq-PA-6 (µm)	Ra- ABS (µm)	Rq-ABS (µm)	Ra-PA-6 (µm)	Rq-PA-6 (µm)	Ra-ABS	Rq-ABS	Ra-PA-6	Rq-PA-6
2	3.004	3.694	21.652	26.61	3.017	3.696	21.646	26.647	0.45	0.05	0.13	0.13
4	3.788	4.924	22.183	27.251	3.791	4.761	22.141	27.205	0.07	3.31	0.67	0.16
6	3.215	3.922	22.367	27.543	3.219	3.914	22.348	27.533	0.15	0.20	0.37	0.03
7	4.112	5.098	22.513	27.518	4.053	5.055	22.525	27.647	1.4	0.84	0.19	0.46
9	2.474	3.043	22.862	27.883	2.543	3.115	22.851	27.976	2.7	2.36	0.19	0.33

## Appendix A

**Table A1:** Mean S/N ratio response table for Ra-ABS polymer.

Level	A (mm)	B (mm)	C (°C)	D	E (°C)	F (mm/s)	G (mm)
1	−10.128	**−9.305**	−10.386	**−9.658**	−10.496	**−9.347**	**−8.471**
2	**−9.930**	−10.124	−10.055	−10.001	−10.022	−9.999	−10.158
3		−10.658	**−9.646**	−10.428	**−9.569**	−10.740	−11.459
Delta	0.198	1.352	0.740	0.770	0.927	1.393	2.988
Rank	7	3	6	5	4	2	1

**Table A2:** Mean S/N ratio response table for Rq-ABS polymer.

Level.	A (mm)	B (mm)	C (°C)	D	E (°C)	F (mm/s)	G (mm)
1	−11.93	**−11.10**	−12.32	**−11.52**	−12.36	**−11.12**	**−10.27**
2	**−11.86**	−12.04	−11.88	−11.94	−11.86	−12.00	−12.00
3		−12.55	**−11.49**	−12.22	**−11.46**	−12.56	−13.42
Delta	0.07	1.44	0.83	0.70	0.90	1.44	3.15
Rank	7	2	5	6	4	3	1

**Table A3:** Mean S/N ratio response table for Ra-PA-6 polymer.

Level	A1 (mm)	A2 (mm/s)	A3 (°C)	A4
1	**−26.70**	**−26.87**	−26.95	**−26.91**
2	−26.95	−26.95	−26.94	−26.93
3	−27.16	−26.98	**−26.91**	−26.97
Delta	0.45	0.11	0.04	0.05
Rank	1	2	4	3

**Table A4:** Mean S/N ratio response table for Rq-PA-6 polymer.

Level	A1 (mm)	A2 (mm/s)	A3 (°C)	A4
1	**−28.50**	**−28.65**	−28.75	**−28.69**
2	−28.75	−28.74	−28.71	−28.70
3	−28.90	−28.75	**−28.69**	−28.75
Delta	0.40	0.11	0.05	0.06
Rank	1	2	4	3

**Table A5:** The Confirmation test outcomes for Ra-ABS and Rq-ABS polymer.

	Initial Parameters		Optimal Parameters	
	Prediction	Experimental	Prediction	Experiment
**Level**	A-S2 B-S2 C-S2 D-S2 E-S2 F-S2 G-S2	A-S2 B-S2 C-S2 D-S2 E-S2 F-S2 G-S2	A-S2 B-S1 C-S3 D-S1 E-S3 F-S1 G-S1	A-S2 B-S1 C-S3 D-S1 E-S3 F-S1 G-S1
**Ra (µm)**		3.259		1.753
**Rq (µm)**		4.078		2.073
**S/N ratio (dB) for Ra (µm)**	−10.280	−10.275	−5.754	−5.801
**S/N ratio (dB) for Rq (µm)**	−12.207	−12.20	−7.45	−7.463
**Improvement in S/N ratio (dB) for Ra (um)**	4.474			
**Improvement in S/N ratio (dB) for Rq (um)**	4.737			
**% Reduction of Ra-ABS**	85.99%			
**% Reduction of Rq-ABS**	96.7%			

**Table A6:** The Confirmation test outcomes for Ra-PA-6 and Rq-PA-6 polymer.

	Initial Parameters		Optimal Parameters	
	Prediction	Experimental	Prediction	Experiment
**Level**	A1-S2 A2-S2 A3-S2 A4-S2	A1-S2 A2-S2 A3-S2 A4-S2	A1-S1 A2-S1 A3-S3 A4-S1	A1-S1 A2-S1 A3-S3 A4-S1
**Ra (µm)**		22.25		21.37
**Rq (µm)**		27.378		26.24
**S/N ratio (dB) for Ra (µm)**	−26.96	−26.95	−26.5971	−26.60
**S/N ratio (dB) for Rq (µm)**	−28.7482	−28.740	−28.377	−28.366
**Improvement in S/N ratio (dB) for Ra (µm)**	0.36			
**Improvement in S/N ratio (dB) for Rq (µm)**	1.138			
**% Reduction of Ra-PA-6**	4.87%			
**% Reduction of Rq-PA-6**	4.33%			
